# Utilization of the Lateral Extracavitary Approach for the Resection of a Giant Extradural Paraspinal Schwannoma: A Case Report

**DOI:** 10.1227/neuprac.0000000000000108

**Published:** 2024-09-26

**Authors:** Khashayar Mozaffari, Max Fleisher, Peter Harris, Michael K. Rosner

**Affiliations:** Department of Neurological Surgery, The George Washington University Hospital, Washington, District Of Columbia, USA

**Keywords:** Lateral extracavitary approach, Paraspinal schwannoma, Spine surgery

## Abstract

**BACKGROUND AND IMPORTANCE:**

Spinal schwannomas are benign neoplasms originating from the spinal nerve sheath and account for around one-third of primary spine neoplasms. The most common treatment modality for these tumors is complete surgical resection. Compared with intradural tumors, the resection of an extradural spinal schwannoma is generally associated with a more complex approach, including longer incisions and increased lateral exposure. One useful surgical technique is the lateral extracavitary approach (LECA), which enables dorsal and ventrolateral access to the thoracolumbar spine with decreased rates of morbidity. Herein, the authors describe this approach for the resection of a giant extradural paraspinal schwannoma.

**CLINICAL PRESENTATION:**

A 74-year-old female patient presented with right flank pain and difficulty breathing during strenuous exercise. Imaging revealed a large 8.5 × 5.2 × 6.3 cm solid paraspinal lesion spanning from T11-L2 vertebral body levels, with mass effect on the right posterior diaphragm and lung. Before surgical resection, the lesion was confirmed to be a schwannoma by needle biopsy. A LECA approach was used, achieving gross total resection. At 1-month follow-up, the patient reported great symptomatic resolution.

**CONCLUSION:**

LECA proved to be an instrumental approach in a technically challenging resection of a giant extradural paraspinal schwannoma.

**ABBREVIATION:**
LECA: lateral extracavitary approach.

Spinal schwannomas are benign tumors that arise from the peripheral nerve sheath of the spinal cord.^1-3^ The majority of these tumors are intradural, and approximately 15% include an extradural component, and rare cases are purely extradural.^2,4-6^ Tumors vary in size, and according to the classification by Sridhar et al,^7^ those spanning multiple vertebral segments, alongside extraspinal components, constitute “giant spinal schwannomas.”

The “gold standard” for spinal schwannomas is complete surgical resection.^3,8^ While surgical approach depends on the anatomic location of the tumor, extradural schwannomas generally require a complex approach, longer incisions, and increased lateral exposure.^5^ Specifically, the lateral extracavitary approach (LECA) is a commonly used approach that allows for dorsal and ventrolateral access to the thoracolumbar spine with decreased rates of morbidity.^9^ While originally described by Larson et al in 1976 for trauma cases, this approach addresses a wide range of pathologies including tumors, infection, disk herniation, and deformity.^9,10^ Herein, the authors describe using LECA for the resection of a giant extradural paraspinal schwannoma. Although not a unique approach for this pathology, there is a paucity of literature on these tumors in general and far fewer operative videos demonstrating the utilization of this technique for this rare pathology.

## CLINICAL PRESENTATION

**FIGURE 1.**
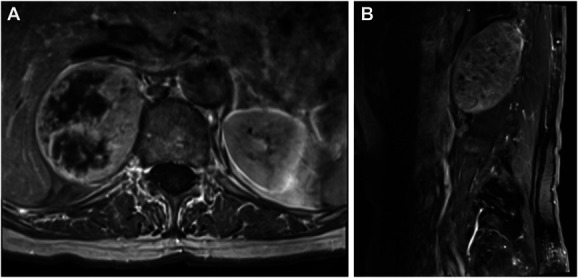
Preoperative T1-MRI with contrast demonstrating a heterogeneously enhancing lesion measuring 8.5 × 5.2 × 6.3 cm in **A**, axial and **B**, sagittal views, spanning T11-L2 vertebral bodies.

A 74-year-old female patient presented with right flank pain and difficulty breathing during strenuous exercise. She had a history of breast cancer that was diagnosed 20 years prior and treated with mastectomy and adjuvant chemoradiation, with no evidence of recurrence since initial therapy. The patient was first referred to endocrinology for a suspected “adrenal nodule,” although follow-up imaging revealed an extremely large 8.5 × 5.2 × 6.3 cm solid paraspinal lesion (Figure 1). A needle biopsy further characterized the lesion as a homogeneous benign, yet necrotic paraspinal nerve sheath schwannoma with an additional mass effect on the right posterior diaphragm and lung. The patient consented to the procedure and publication of all materials, and our institution does not require Institutional Review Board approval for the presentation of a single case.

**FIGURE 2.**
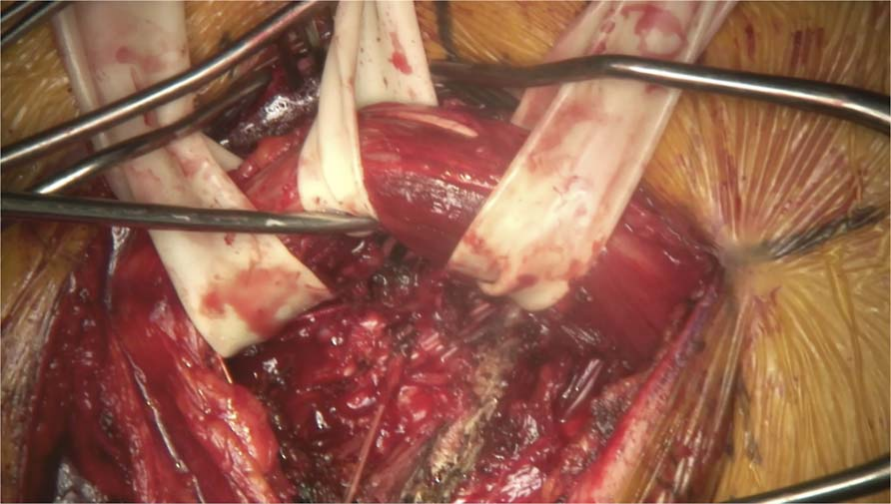
Utilization of the penrose drain to isolate multifidus and spinalis muscles.

**FIGURE 3.**
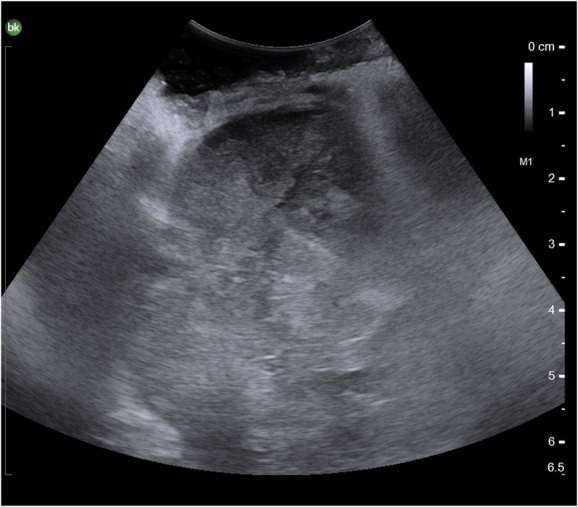
Intraoperative ultrasound for visualization of the tumor margins.

A posterior thoracolumbar lateral extracavitary exposure approach was used, a variation of the aforementioned LECA procedure (**Supplemental Digital Content 1**, http://links.lww.com/NS9/A32). Fluoroscopy was used to localize the correct levels of the thoracolumbar spine and plan the incision. A J-shaped or “hockey stick” incision was marked accordingly to expose the right paraspinous musculature. A myocutaneous flap including the latissimus dorsi was reflected laterally, exposing spinous processes and the paraspinous muscles. Multifidus and spinalis were dissected from midline and the more lateral erector spinae muscles, respectively, using electrocautery. These were isolated with the aid of penrose drains as depicted in Figure 2 to facilitate mobilization when needed. Further dissection was completed on the right side from T10-L2, exposing the midline as well as laminae on the right, while preserving the facet capsules. Attention was then directed towards T11 and T12 ribs which were exposed distally approximately 7 cm. Fluoroscopy once again confirmed the appropriate levels. Intraoperative ultrasound was also used at this point to visualize tumor margins (Figure 3) and distinguish it from lung margins as they were similar in appearance. With the erector spinae muscles separated adequately, the T11 and T12 ribs were cut and removed with straight osteotomes to expose the margins of the tumor.

The well-circumscribed mass was thus further exposed and was reddish gray and highly vascularized. Microdissection instruments were used to complete dissection, keeping the capsule and surrounding structures intact. Owing to the size of the mass, internal debulking was carried out with ultrasonic aspiration. The capsule was traced back to the T11 nerve root, which was isolated and cut. Sharp and blunt microdissection defined the border with cottonoids protecting normal tissue planes, splenic vessels, and thoracic duct, which were deep to the mass, and the thin surrounding pleura. The distal end of the nerve exiting from the mass was identified and cut. The mass was completely removed and sent for permanent specimen (Video). Hemostasis was obtained. No residual elements were noted. The thinned pleura was inspected, and a small pleurotomy was repaired primarily. Valsalva maneuver demonstrated no bubbles through copious antibiotic-impregnated irrigation. Hemovac drains were secured subfascially. Fascial and subcutaneous layers were closed with dissolvable sutures. Operative time was 5 hours and 30 minutes.

Chest x-ray in the setting of increased work of breathing on postoperative day 1 demonstrated hemothorax, which was managed with a thoracostomy tube, and the patient was observed in the intensive care unit for 24 hours. Her hospital course was otherwise unremarkable, and she was discharged home on postoperative day 5. Histopathology analysis confirmed the diagnosis of schwannoma. At the 9-month follow-up visit, the patient reported symptomatic resolution and was neurologically intact, and imaging demonstrated no recurrence.

## DISCUSSION

Purely extradural spinal schwannomas are extremely rare and are associated with a more complex surgical approach.^4,5^ Furthermore, tumors extending over more than 2 vertebral levels are classified as giant lesions, posing a unique challenge due to their size.^7^ Herein, the authors described the utilization of the LECA approach for gross total resection of a giant purely extradural paraspinal schwannoma.

Our patient presented with radicular pain, which is a classic presentation of these lesions.^11,12^ The mass spanned across T11-L2 vertebral bodies, measuring 8.5 × 5.2 × 6.3 cm, exerting mass effect on the right posterior diaphragm and lung, along with proximity to several other anatomic structures including the thoracic duct. These features along with its purely extradural nature contributed to the complexity of this case. We used neuromonitoring in the form of motor-evoked potentials and somatosensory-evoked potentials, and there were no changes throughout the duration of the case. One can also consider triggered electromyography if there is any doubt regarding proximal and distal attachments or if the function of the nerve in question would change the operative plan.

In this case, the LECA approach provided dorsal and ventrolateral access to the spine with a more favorable complication profile compared with the traditional transpleural and transperitoneal approaches which harbor a great risk of retraction injury.^9^ Alternatives including an anterior or anterolateral approach would have been significantly limited by the proximity to the liver. By approaching the mass from the ventrolateral corridor, manipulation of the liver and great vessels is minimized. Other options were the midline approach which would have limited the lateral exposure and thus visualization of the entire lesion. More lateral approaches would have been hindered by the tumor's spatial relationship with the lung. In addition, LECA provides access without any spinal cord manipulation, and if needed, it allows for posterior instrumentation. In cases of giant thoracolumbar schwannomas, the anatomic relationship with the lungs, diaphragm, kidneys, the thoracic duct, splenic and renal vessels are all unique considerations that need to be carefully studied before operating.

Although LECA offers several advantages, surgeons and patients must be aware of its risks. As noted in a systematic review by Foreman et al,^9^ LECA is inherently more susceptible to pulmonary complications compared with traditional posterior approaches. We encountered an intraoperative pleurotomy which was repaired primarily and a small hemothorax which was managed with a thoracostomy tube. The adherent nature of the tumor to the diaphragm and right lung likely contributed to this complication. Use of ultrasound to define tumor borders and meticulous debulking before pulling the tumor capsule toward the center of the resection cavity can help reduce risk of retraction injury and violating normal anatomical border in future cases similar to ours. Although our patient had a benign hospital course, such potential complications need to be considered in preoperative planning, particularly in patients with preexisting lung disease.

## CONCLUSION

We presented the utilization of LECA for gross total resection of a giant purely extradural paraspinal schwannoma of the thoracolumbar spine. This case demonstrates the unique advantages of this approach for tumor visualization while protecting the surrounding anatomic structures. However, despite its advantages, surgeons must take into consideration the greater risk of pulmonary complications with LECA compared with traditional posterior approaches. Nonetheless, this case advocates for the application of this technique for tackling complex pathologies of the spine.

## SUPPLEMENTAL DIGITAL CONTENT

**Supplementary Figure 1**. Operating room setup, workflow, and equipment used.
